# Nutritional Treatment in a Cohort of Pediatric Patients with Familial Hypercholesterolaemia: Effect on Lipid Profile

**DOI:** 10.3390/nu14142817

**Published:** 2022-07-08

**Authors:** Maria Elena Capra, Cristina Pederiva, Claudia Viggiano, Enrico Fabrizi, Giuseppe Banderali, Giacomo Biasucci

**Affiliations:** 1 Centre for Paediatric Dyslipidaemias, Paediatrics and Neonatology Unit, Guglielmo da Saliceto Hospital, 29121 Piacenza, Italy; m.capra@ausl.pc.it (M.E.C.); g.biasucci@ausl.pc.it (G.B.); 2 Department of Translational Medical and Surgical Sciences, University of Parma, 43126 Parma, Italy; 3 Clinical Service for Dyslipidaemias, Study and Prevention of Atherosclerosis in Childhood, Paediatrics Unit, ASST-Santi Paolo e Carlo, 20142 Milan, Italy; claudia.viggiano@unimi.it; 4 DISES & DSS, Università Cattolica Del S. Cuore, Via Emilia Parmense 84, 29122 Piacenza, Italy; enrico.fabrizi@unicatt.it

**Keywords:** nutrition, familial hypercholesterolaemia, children, lipid profile, diet, lifestyle

## Abstract

Background and aims: Familial Hypercholesterolaemia (FH) is characterised by a genetic alteration in the transport and metabolism of cholesterol that leads to elevated levels of total cholesterol (CT) and low-density lipoprotein cholesterol (LDL-C) and early onset of atherosclerosis. According to the current guidelines, diet and promotion of healthy habits are first-line treatments. Little is known about the effectiveness of cholesterol-lowering diet and healthy lifestyle habits on plasma cholesterol and lipid profile in children and adolescents with FH. The aim of the study is to investigate the effect of the nutritional counseling on plasma lipid profile in FH children at the first step of treatment. Methods: 115 FH children (2–17 years) were included in the study; dietary habits were evaluated through a Food Frequency Questionnaire (FFQ) and blood samples for lipid profile were collected at the enrollment (T0) and six months later (T1). Results: the lipid profile at T0 and T1, expressed as mean ± standard deviation in mg/dL, was, respectively: total cholesterol 285.9 ± 51.1 and 276.6 ± 46.8 (paired test difference *p* value < 0.01), LDL-cholesterol 214.9 ± 47.7 and 206.4 ± 46.6 (*p* value < 0.01), HDL-cholesterol 52.9 ± 13 and 54.4 ± 11.5 (*p* value 0.07), triglycerides 87 ± 46.7 and 82.2 ± 38.4 (*p* value 0.4), non-HDL cholesterol 233 ± 51.4 and 222.2 ± 47.4 (*p* < 0.01). In the dietary habits (weekly portions) we observed an improvement (*p* ≤ 001) for fruit and vegetables, fish, pulses, whole foods, and a reduction (*p* < 0.01) for meat, sausages, cheese, junk foods consumption. Conclusions: In FH children we have highlighted an improvement of the plasma lipid profile and in healthy dietary habits after nutritional counseling.

## 1. Introduction

Familial Hypercholesterolaemia (FH) is a genetic disease characterised by an alteration in the transport and metabolism of cholesterol. It is a common genetic cause of premature coronary artery disease (CHD). FH is an autosomal dominant genetic disorder, except for a rare autosomal recessive form. In most cases, FH is caused by a mutation in the LDL receptor gene (LDL-R gene), resulting in absent or dysfunctional receptors on the surface of hepatocytes and a reduced liver capacity to remove atherogenic cholesterol-rich low-density lipoproteins (LDLs) from the circulation, with consequent accumulation of LDL cholesterol (LDL-C). Constant exposure of the arterial walls to high levels of LDL-C accelerates the deposition of cholesterol with the development of atherosclerotic lesions, causing the early onset of atherosclerosis. The prevalence of the heterozygous FH is 1 per 200–250 subjects in the general population [1].

Treatment of FH is a lifelong process: early intervention can reduce the negative effect of high LDL-C levels, improving endothelial function and reducing atherosclerosis development [2,3,4]. Diet and promotion of healthy habits are considered as first-line treatments. Patients and their families should undergo education targeting lifestyle management and should be informed about food choices [5]. The aim of this approach is to teach children and their families correct nutritional habits that can become a lifelong habit and last until adulthood [6]. Parents of children with hypercholesterolaemia often underestimate its effects as well as the CHD issue; as a consequence, thorough nutritional and lifestyle counseling for the whole family is an issue of utmost importance [7].

Dietary factors may influence the pathogenesis of atherosclerosis directly or through the prevention of the development of adiposity, elevated blood pressure effects and hyperglycemia. Total cholesterol (TC) and LDL-C levels can be modulated by dietary fatty acids and cholesterol intake. The lipid intake, in particular cholesterol and saturated fats, is a major determinant of blood cholesterol levels [6]. A low lipid diet, including a restriction of saturated fats intake <7% of total calories and daily cholesterol intake <200 mg, is recommended. The intake of whole grains, low-fat dairy products, beans, fish, lean meats, fruit, and vegetables should be encouraged. The ideal diet should present the following features: protein intake of 12–14% of total daily calories (animal/vegetable protein ratio of 1:1); carbohydrates intake of 55–60% of total daily calories, lipid intake below 30% but above 25% of total daily calories, subdivided in saturated <7%, monounsaturated 10–15%, and polyunsaturated fats 5–10% [8,9].

Little is known about the effectiveness of cholesterol-lowering diet and healthy lifestyle habits on plasma cholesterol and lipid profile in children and adolescents with FH. The aim of the study is to characterise FH children’s’ diet and habits and to investigate the effect of the nutritional counseling on their plasma lipid profile at the first step of treatment.

## 2. Materials and Methods

### 2.1. Study Design and Population

This is a retrospective, observational study on good clinical practice involving two Lipid Centers in Northern Italy. The aim of the study is to evaluate the effect of nutritional treatment on lipid profile in a cohort of paediatric patients with severe hypercholesterolaemia, visited for the first time at the Lipid Paediatric Centre of Piacenza Guglielmo da Saliceto Hospital and Milan San Paolo Hospital.

Eligibility criteria were: clinical or genetic diagnosis of FH; age 2–17 years, patients visited for the first time in the above mentioned paediatric lipid centres, and a written informed consent obtained from parents and/or caregivers. Exclusion criteria were secondary causes of hypercholesterolaemia (altered thyroid function, liver and/or kidney disease, pharmacological treatment that can alter lipid profile as a side effect); mild hypercholesterolaemia with LDL-C < 160 mg/dL without positive family history for premature coronary heart disease and/or hypercholesterolaemia in first degree relatives; and lipid profile alterations other than hypercholesterolaemia. According to EAS guidelines [1], positive family history for premature coronary heart disease is defined as the presence of angina, heart stroke, percutaneous coronary angioplasty and/or coronary bypass below 55 years of age in male subjects and lower than 60 years in female subjects.

**Figure 1:**
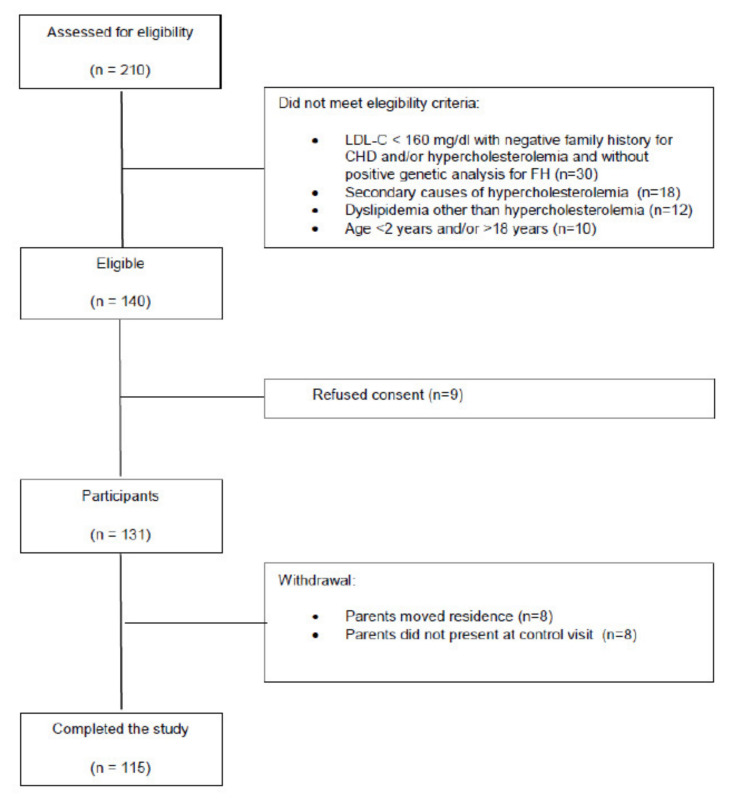
Progression of patients through the study.

Over a three year period (May 2015–May 2018), 210 patients were assessed for eligibility in the Piacenza Guglielmo da Saliceto Hospital and Milan San Paolo Hospital; 140 patients were eligible for the study; nine patients refused to participate; 16 patients withdrew from the study and 115 patients completed the study. The flowchart of subjects’ enrollment is shown in Figure 1.

### 2.2. Study Variables

At enrollment, detailed cardiovascular disease-oriented and dyslipidaemia family history in first and second degree relatives was collected. At T0 (enrollment) and T1 (after six months), all patients were evaluated for anthropometric measures. Body Mass Index (BMI) was expressed as kilograms of body weight/m^2^ height ratio, wearing just underwear and using standard equipment. Systolic and diastolic blood pressure were also measured. Paediatric evaluation with assessment of pubertal status according to the Tanner stage was performed. All patients had blood samples drawn at enrollment (T0) and after six months (T1). Blood samples were collected after 12-h overnight fast and in good clinical conditions. Serum total cholesterol, LDL-cholesterol, HDL-cholesterol, triglycerides, apolipoprotein A1, apolipoprotein B (enzymatic colorimetric assay), lipoprotein(a) (immunoturbidimetric assay), and homocysteine were assayed. Non-HDL-cholesterol was calculated as TC minus HDL-cholesterol. Genetic analysis for familial hypercholesterolaemia was performed by means of DNA extraction from blood samples after obtaining written informed consent from parents and/or caregivers. 

Physical and sport activity were evaluated by asking each patient how many hours that he/she spent every week practicing physical and/or sport activities. Daily screen time hours (TV/tablet/mobile phone/video-games) were investigated as well, excluding the use of screen devices for school and/or study purposes. 

Nutritional habits were evaluated by a Paediatric Dietician through the admission of a food frequency questionnaire (FFQ), and tailored nutritional advice was given to each patient according to STEP one indications and to the analysis of each FFQ.^5^ FFQ is a semiquantitative questionnaire analysing the frequency of assumption of 113 food items that is validated for pediatric age [10]. The FFQ included questions about the consumption of various type of foods during the past year. The size of the typical portion and the consumption frequency of each item had to be specified. The use of FFQ has been validated in the paediatric population in mediterranean countries [10].

FFQ was self-administered during medical evaluation in order to minimise bias, and was analysed by a paediatric dietician. For patients aged less than 14 years, parents or caregivers completed the FFQ, whereas patients aged 14 to 17 years old completed the questionnaire on their own in the presence of a parent/caregiver. Nutritional tailored counseling was then given to the child/adolescent in the presence of the parent/caregiver.

Lipid profile, nutritional and lifestyle habits were evaluated after six months according to good clinical practice. None of the participants were on drug therapy and none of them were given nutraceuticals or food supplements before or during the study.

### 2.3. Statistic Analysis

Signed-rank Wilcoxon tests for paired samples were used to compare patients before and after the nutritional treatment. Comparisons have been conducted both for the sample as a whole and separately for the patients with or without positive family history for premature coronary heart disease. For 0/1 variables the pre-post comparisons are based on a McNamar’s test. Statistical significance was set at *p* ≤ 0.01.

## 3. Results

General characteristics of the study population are shown in Table 1. Male and female patients were not different at baseline for general characteristics.

**Figure 2:**
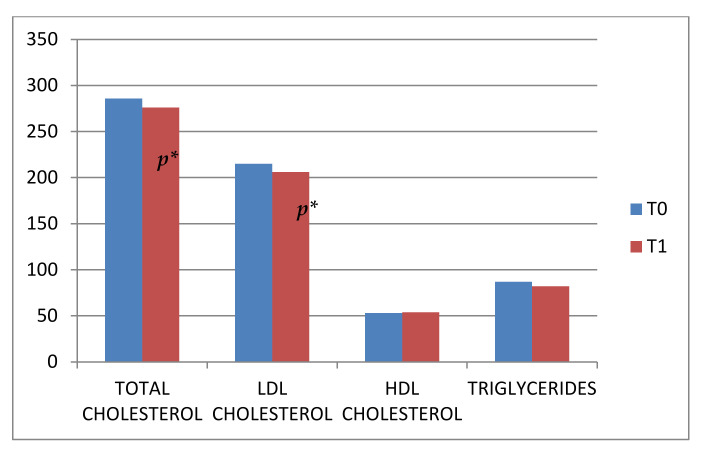
The percentage changes in total cholesterol, low-density lipoprotein cholesterol (LDL-C), high-density lipoprotein cholesterol (HDL-C), and triglyceride concentrations at T0 and T1. * Statistical significance is considered at a *p* value ≤ 0.01. Data are expressed in mg/dL.

The lipid profile at T0 and T1, expressed as mean ± standard deviation in mg/dL, was: total cholesterol 285.9 ± 51.11 and 276.6 ± 46.8 (Wilcoxon paired test on the difference between distributions yields *p* < 0.01), LDL-cholesterol 214.9 ± 47.7 and 206.4 ± 46.6 (*p* < 0.01), HDL-cholesterol 52.9 ± 13 and 54.4 ± 11.1 (*p* = 0.07), triglycerides 87 ± 46.7 and 82.2 ± 38.4 (*p* = 0.4), non-HDL cholesterol 233 ± 51.4 and 222.2 ± 47.4 (*p* < 0.01). The lipid profile was different after nutritional and lifestyle intervention, as shown in Figure 2. 

Most dietary habit items analysed were significantly different at T0 and T1, as shown in Table 2. 

**Figure 3:**
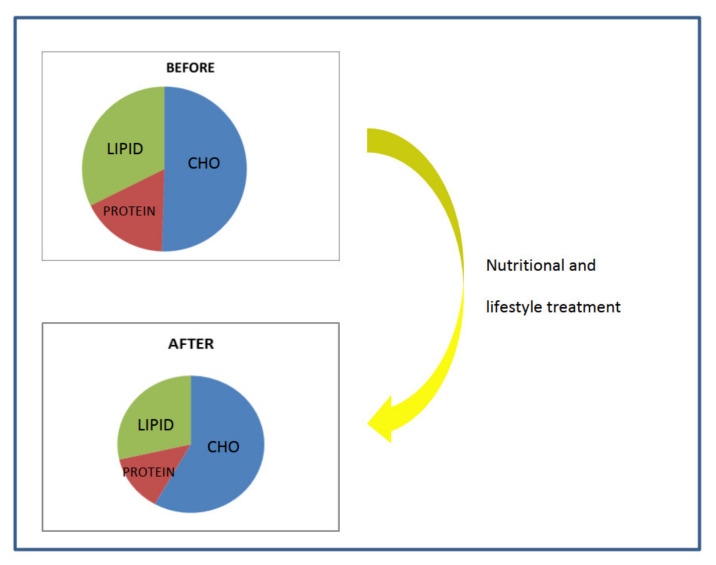
Macronutrients intake of the study population before and after nutritional and lifestyle intervention.

There was no difference in breakfast consumption. Macronutrient intake was significantly modified after nutritional and lifestyle intervention, as shown in Table 3 and in Figure 3. 

Physical activity weekly hours, expressed as mean ± standard deviation, were 1.6 ± 0.7 at T0 and 1.63 ± 0.3 at T1, with a *p* of 0.11. Sport weekly activity hours, expressed as mean ± standard deviation, were 1.4 ± 1.5 at T0 and 1.4 ± 1.5 at T1, with a *p* value of 0.02. Screen time daily hours were 2.7 ± 1.5 at T0 and 2.5 ± 1.5 at T1 with a *p* value < 0.01. Regression logistic analysis confirmed these findings.

Focusing on CHD family history, lipid profile is shown in Table 4 and Table 5.

The comparison of the two groups of patients with or without positive family history for cardiovascular disease with respect to their lipid profile at baseline (T0), conducted by means of signed-rank Wilcoxon tests, did not provide evidence of significant differences for each of the involved variables.

## 4. Discussion

Our study has highlighted an improvement both in lipid profile and in nutritional habits in children and adolescents with FH after nutritional treatment. Focusing on lipid profiles after nutritional intervention, we have observed a reduction in all atherogenic lipid components (TC, LDL-C and non-HDL-C) that are closely linked to an increased atherosclerosis risk, while HDL cholesterol levels are slightly increased and triglycerides levels are not significantly modified. Our results are consistent with those reported by the first authors [11] that have analysed the variation in lipid profile in relation to nutritional habits. These authors [12,13,14] reported an average reduction of 6 to 10% of LDL-cholesterol levels after nutritional intervention. Tonstad et al. [15] reported that the lipid profiles of children with FH are not dependent on dietary lipid intake, but that nutritional treatment can modify plasma lipid values of children with FH. In the DISC study [16], one of the largest randomised controlled clinical trials on the efficacy and safety of dietary intervention to reduce LDL-cholesterol levels in pre-pubertal children, an average LDL-cholesterol reduction of 11.8% was reported in a population of children with medium-high basal lipid levels (LDL-cholesterol about 95° centile for age and sex). More recent studies [17,18] have reported less consistent results [19,20,21]. In the Cochrane review published in 2010 [22], the authors stated that there is not enough evidence on the efficacy of cholesterol lowering diet on lowering lipid values in a subject with FH. In a recent meta-analysis [23] looking at the impact of diet on lipid profile in patients with FH, a total lack of efficacy of nutritional intervention on LDL-cholesterol values was reported. In this meta-analysis, the authors hypothesised that this result is mainly determined by biases in study designs rather than by a true lack of effect.

In the last decade, mendelian randomization studies [24] have validated the role of LDL-cholesterol on the pathogenesis of atherosclerosis, not only in terms of elevated absolute values, but also in terms of arterial wall cumulative exposure to LDL-cholesterol, the so called “LDL-burden” [1]. Moreover, a slight reduction in LDL-cholesterol plasma values, when maintained for life, has been proven to be responsible for an important reduction (up to 53%) of coronary-heart disease [25]. In this context, the moderate reduction in total and LDL-cholesterol observed in our study can have a relevant clinical impact, especially if maintained throughout adolescence and adulthood. In our study, HDL-cholesterol and triglycerides levels were not significantly influenced by diet. This finding is not consistent with the results reported in previous studies [16,17,18], that, on the contrary, highlighted a reduction of HDL-cholesterol in response to diet. We speculate that this difference may be due to a more balanced and not too restrictive nutritional intervention. Triglycerides levels were stable before and after nutritional treatment, suggesting that there was no excessive consumption of simple carbohydrates and good fiber consumption in our population.

When we decided to analyse our study population according to family history for premature CHD, we thought that patients belonging to families with positive family history for premature CHD would have shown a better response to nutritional and lifestyle treatment in terms of lipid profile improvement. In fact, patients with positive family history for premature CHD belong to a family environment already CHD problem oriented. However, our data show that in patients with a positive family history for premature CHD, lipid profiles are improved after nutritional and lifestyle intervention, but this is only a tendency that does not reach statistical significance in our sample. This report may reflect the fact that families that have already experienced premature CHD often put into action nutritional restrictions even before medical counselling in a specialised centre, as parents often tend to make their offspring follow their own dietetic patterns.

As reported in Table 2, the nutritional habits of our population were already good at T0: 92% of subjects regularly had breakfast, fruit and vegetables were consumed at least twice a day (16 portions per week), meat and cheese intake only slightly exceeded the advisable four weekly portions, and whole foods were generally already present in the subjects diets. This indicates that, even before specific nutritional counseling has been given, nutritional habits had already been modified by the parents, considering that children and adolescents with FH generally have an affected relative in their family. However, in our study we have reported an improvement in food choices and in nutritional habits after nutritional counseling; in particular, we observed a reduction in fat-rich foods (meat, cheese and various dressings) and an increase in pulses, fish (rich in omega 3 polyunsaturated fatty acids) and whole foods (rich in fibres). We also reported an increase in fruit and vegetables intake, a slight increase in egg intake (proposed as a “possible food” once a week), and an important reduction in junk food consumption. Our results are consistent with those reported in the DISC study [16], showing a good percentage of consumption of “go” (less atherogenic) foods in respect to that of “whoa” foods (more atherogenic) at basal time, both in the intervention and in the control group. In the DISC study, after nutritional intervention, the intake of “go foods” was improved both in the intervention (67.4% of total energy) and in the control group (56.8% of total energy) [16,26]. Cicero et al. [18] analysed a cohort of children with FH. They highlighted that a qualitative nutritional counseling, if compared to a quantitative one, not only improves general macro-nutrients consumption but also grants a lower allostatic load and a better compliance. As previously stated, currently published systematic reviews are not conclusive on the efficacy of nutritional intervention on improving lipid profiles in children with FH [22,23].

The present study has a few limitations, the most important of which is its observational nature. Nevertheless, our study included children treated in regular clinical practice, and the results are more consistent with everyday life. Another limitation is that the two lipid centres involved are located in the same geographical region (the North of Italy), so there might be possible local contaminations of the Mediterranean Diet with respect to other Italian locations. Another potential point of concern is the use of FFQ as a tool to evaluate nutritional habits. The FFQ could have some bias, which we tried to limit by proposing an auto-administration of the questionnaire during the paediatric visit with immediate delivery so as to minimise the possible correction to the actual answers. Moreover, we gave each patient tailored nutritional advice without giving standard written nutritional information so as to give better accuracy for the subsequent FFQ administration [27]. 

One of the strengths of our study is the relevant number of the subjects recruited. This, together with the statistically significant effects evaluated both on lipid profile and on nutritional habits after nutritional counseling, validates the importance of tailored and targeted nutritional counseling for children and adolescents with FH. Tailoring of the nutritional counseling is another positive hallmark of our study. On the one hand, tailored counseling is much more time consuming for the operator, but it seems to grant a better compliance both in the short and in the long run. Finally, we want to highlight that lipid profile improvement has been determined only by nutritional and lifestyle treatment, as patients have not been treated with nutraceuticals or food supplements. Moreover, we must not forget that our intervention was meant to help patients reach nutritional and lifestyle habits that have a good chance to last throughout childhood and into adulthood. According to current guidelines, adequate pharmacological therapy might be eventually added to nutritional and lifestyle treatment, if necessary.

## 5. Conclusions

In conclusion, despite the main current documents [1,3,28,29] indicating nutritional treatment as a milestone, when dealing with paediatric subjects with FH, there are still a few studies on this topic, and current evidence is often contrasting. With respect to this aspect, the results of our study support the efficacy and the feasibility of nutritional intervention in children and adolescents with FH as a first step treatment before eventually starting pharmacological therapy.

## Figures and Tables

**Table 1:** General characteristics of the study population.

	All Patients	Male	Female
Number	115	57	58
Age (years, mean ± sd)	9.40 ± 4.11	9.40 ± 3.40	9.31 ± 4.62
BMI (*n*, %)			
1: normal weight	82 (71.30%)	40 (70.17%)	42 (72.41%)
2: overweight	18 (15.65%)	9 (15.79%)	9 (15.50%)
3: obese	11 (9.58%)	6 (10.52%)	5 (8.62%)
4: underweight	4 (3.47%)	2 (3.51%)	2 (3.47%)
Genetic analysis for FH (positive, %)	85 (73.91%)	43 (75.43%)	42 (72.41%)
Total cholesterol (mg/dL, mean ± sd)	285.87 ± 51.11	281.19 ± 55.78	290.46 ± 46.09
LDL cholesterol (mg/dL, mean ± sd)	214.94 ± 47.73	210.72 ± 51.07	219.08 ± 44.27
HDL cholesterol (mg/dL, mean ± sd)	52.85 ± 13.10	53.02 ± 12.26	52.70 ± 13.72
Triglycerides (mg/dL, mean ± sd)	87.03 ± 46.75	78.23 ± 34.18	95.67 ± 55.32
Non-HDL cholesterol (mg/dL, mean ± sd)	233.02 ± 51.44	228.19 ± 55.21	237.76 ± 47.40

**Table 2:** Dietary and lifestyle habits of the study population at baseline (T0) and after six months of nutritional and lifestyle intervention (T1).

	T0	T1	*p Value*
**Breakfast (yes, *n*, %)**	106 (92%)	109 (95%)	0.37
Fruit and vegetables (weekly portions, mean ± sd)	16.0 ± 11.4	22.3 ± 10.1	<0.01 *
Meat (weekly portions, mean ± sd)	4.5 ± 2.4	3.7 ± 1.2	<0.01 *
Sausages (weekly portions, mean ± sd)	2.7 ± 1.9	1.6 ± 1.2	<0.01 *
Fish (weekly portions, mean ± sd)	1.8 ± 1.2	2.4 ± 1.1	<0.01 *
Pulses (weekly portions, mean ± sd)	1.5 ± 1.6	1.8 ± 1.2	0.01 *
Cheese (weekly portions, mean ± sd)	2.9 ± 2.3	1.5 ± 1.3	<0.01 *
Eggs (weekly portions, mean ± sd)	0.7 ± 0.7	0.9 ± 0.6	0.01 *
Whole foods (weekly portions, mean ± sd)	1.4 ± 3.5	2.6 ± 4	<0.01 *
Junk foods (weekly portions, mean ± sd)	8.2 ± 6.5	4.3 ± 3.6	<0.01 *
Screen time (daily hours, mean ± sd)	2.7 ± 1.5	2.5 ± 1.5	<0.01 *
Physical activity (daily hours, mean ± sd)	1.6 ± 0.7	1.6 ± 0.3	0.11
Sport (weekly hours, mean ± sd)	1.36 ± 1.5	1.42 ± 1.5	0.02

Signed-rank Wilcoxon test for paired samples are used to compare patients before and after the nutritional and lifestyle treatment. For 0/1 variables the pre-post comparisons are based on a McNamar’s test. * Statistical significance is considered as *p* value ≤ 0.01.

**Table 3:** Macronutrients intake of the study population at baseline and after six months.

	T0	T1	*p* Value
Carbohydrates (Energy %)	50.1 ± 2.3	57.2 ± 2.7	<0.01 *
Fiber (g/day)	3.8 ± 2.4	5.5 ± 1.2	0.08
Protein (Energy %)	17.3 ± 1.6	13.8 ± 1.2	<0.01 *
Fat (Energy %)	32.8 ± 2.3	29.3 ± 2.5	<0.01 *

A signed-rank Wilcoxon test for paired samples was used to compare patients before and after the nutritional and lifestyle treatment. Energy %, percentage of energy. Values are mean and SD, * Statistical significance set at *p* ≤ 0.01.

**Table 4:** Lipid profile of the study population with positive family history for CHD (*n* = 66) at baseline (T0) and after nutritional and lifestyle intervention (T1).

	T0	T1	*p Value*
Total cholesterol	283.47 ± 48.84	279.17 ± 48.03	0.31
LDL cholesterol	214.62 ± 48.69	209.41 ± 47.38	0.23
HDL cholesterol	53.01 ± 12.84	53.65 ± 10.89	0.37
Triglycerides	82.36 ± 39.71	78.15 ± 29.19	0.39
Non-HDL cholesterol	230.47 ± 50.23	225.52 ± 49.80	0.22

Values are expressed in mg/dL, mean ± standard deviation. Signed-rank Wilcoxon test for paired samples are used to compare patients before and after the nutritional and lifestyle treatment. Statistical significance is considered as *p* value < 0.01.

**Table 5:** Lipid profile of the study population without positive family history for CHD (*n* = 49) at baseline (T0) and after nutritional and lifestyle intervention (T1).

	T0	T1	*p Value*
Total cholesterol	289.10 ± 54.37	273.22 ± 45.36	<0.01 *
LDL cholesterol	215.37 ± 46.93	202.43 ± 45.70	<0.01 *
HDL cholesterol	52.65 ± 13.26	55.47 ± 12.31	0.08
Triglycerides	93.31 ± 54.54	87.55 ± 47.93	0.78
Non-HDL cholesterol	236.45 ± 53.31	217.76 ± 44.16	<0.01 *

Values are expressed in mg/dL, mean ± standard deviation. Signed-rank Wilcoxon test for paired samples are used to compare patients before and after the nutritional and lifestyle treatment. Statistical significance is considered as *p* value ≤0.01.

## Abbreviations

CHD: Coronary Health Disease; EAS: European Atherosclerosis Society; FFQ: Food Frequency Questionnaire; FH: Familial Hypercholesterolaemia; HDL: High Density Lipoprotein; LDL: Low Density Lipoprotein; TC: Total Cholesterol.
